# An Evaluation of Muscle Repair Techniques: Implications in Musculoskeletal Healing and Corollaries in Oral-Facial Clefting

**DOI:** 10.3390/jcm10214803

**Published:** 2021-10-20

**Authors:** Jaehoon Kim, Jaehoon Choi, Junhyung Kim, Taehee Jo, Ilseon Hwang, Kihwan Han, Woonhyeok Jeong

**Affiliations:** 1 Dongsan Medical Center, Department of Plastic and Reconstructive Surgery, Keimyung University College of Medicine, Daegu 42601, Korea; pooh920@dsmc.or.kr (J.K.); psjchoi@dsmc.or.kr (J.C.); med69@dsmc.or.kr (J.K.); taeheejo@dsmc.or.kr (T.J.); 2 Dongsan Medical Center, Department of Pathology, Keimyung University College of Medicine, Daegu 42601, Korea; ilseon@dsmc.or.kr; 3 BL Plastic Clinic, Daegu 41938, Korea; khh@dsmc.or.kr

**Keywords:** cleft lip, cleft palate, muscles, suture techniques, facial muscles

## Abstract

We performed an animal study to identify the techniques associated with the best muscle healing outcomes in cleft lip/palate surgery. The right triceps of thirty adult male Sprague–Dawley rats were cut and repaired by three different suture techniques: simple (*n* = 10), overlapping (*n* = 10), and splitting sutures (*n* = 10). Muscle tissues were isolated from 5 rats per group 1 and 8 weeks postoperation. The inflammatory response and muscle fiber healing were evaluated by hematoxylin and eosin (H&E) staining, Western blotting, immunohistochemistry for TNF-α and IL-1β, and immunofluorescence for laminin and MyoD. Grip strength (N/100 g) and spatial gait symmetry were evaluated before surgery and 1, 2, 4 and 8 weeks postoperation. Eight weeks postoperation, grip force per weight was significantly higher in the simple suture (median, 3.49; IQR, 3.28–3.66) and overlapping groups (median, 3.3; IQR, 3.17–3.47) than the splitting group (median, 2.91; IQR, 2.76–3.05). There was no significant difference in range of motion between groups. The simple group exhibited significant remission of inflammation by H&E staining and lower expression of TNF-α and IL-1β than the other groups by Western blotting and immunohistochemistry. Immunofluorescence revealed stronger expression of MyoD and weaker expression of laminin in the splitting group than in the other groups at week 8, indicating prolonged inflammation and healing followed by poor muscle fiber remodeling. Simple and overlapping sutures demonstrated similar functional healing, although greater inflammation and failure to maintain a thicker muscle belly were observed in the overlapping suture group compared with the simple suture group. Therefore, reconstruction of the philtral column with overlapping sutures alone may result in limited long-term fullness, and additional procedures may be needed.

## 1. Introduction

Cleft lips and palates range from complete clefts to hidden clefts, which includes microform cleft lips and submucosal cleft palates. In patients with cleft lip/palate, inappropriate orientation and abnormal insertion of the orbicularis oris muscle (OOM) and/or levator veli palatini (LVP) may be seen. This manifestation is a histological sign of cleft lip and palate, even microform cleft lip and submucosal cleft palate. One important goal of cleft lip/palate repair is closure of muscular diastasis through various surgical techniques [1,2,3]. The philtrum is a unique structure composed of a ridge called the philtral column and a central depression called the philtral dimple. To mimic the shape of the philtral column and dimple in the cleft lip, the cleft surgeon utilizes various muscle suture techniques ranging from interrupted sutures [4] to buried horizontal mattress sutures [5], overlapping sutures [6], coronal splitting sutures [7], interdigitating sutures [8], and splitting sutures with the folding technique [9]. The use of overlapping suturing of the LVP muscle to strengthen the velopharyngeal closure force following palatoplasty has increased in popularity [10]. Indeed, a very large effort has been made to improve surgical outcomes by manipulating muscles in cleft lip/palate surgery.

Because there is limited subcutaneous tissue in the upper lip, including the OOM and subcutaneous tissue, various muscle suture techniques have been reported to reconstruct the philtrum for cheiloplasty. However, there is the question of whether the shape of the muscle could be maintained in the primary form after suturing because muscle is a dynamic structure that constantly contracts and relaxes. A previous study reported that overlapping sutures lost their thickness and stepped shape at the suturing site [11]. Satellite cells are key cells for the maintenance and regeneration of muscle fibers. Satellite cells remain quiescent under normal conditions and differentiate into myoblasts upon muscle injury that fuse and restore damaged muscle [12]. In terms of muscle healing, this might be the most physiological method by which injured muscle fibers can be correctly joined together in an end-to-end manner. Delayed healing could induce scar formation to reduce the tensile strength of sutured muscle [11]. In turn, a reduced tensile strength could impair the muscle strength and be a potential cause of scar contracture, widening and depression. Therefore, our animal study was performed with a focus on the physiological muscle healing achieved by popular muscle suture techniques in cleft lip/palate surgery.

To demonstrate histologic and functional basis upon which muscle wound heals, we used rat triceps muscle rather than orofacial muscles. It is arguable whether use of the triceps muscle of the rat can be rationalized for drawing conclusions in orofacial muscles. However, each procedure from giving sutures on barely visible orofacial muscles to measuring physiologic strength of muscle in animal model is so difficult to be performed that indirect method has been used where the minimum force to separate muscle tissue was measured [11]. Fortunately, wound healing process in orofacial muscle could be predictable based on the known differences between satellite cell dynamics in somite-derived muscles and those in cranial mesoderm-derived muscles. Cranial mesoderm-derived muscles have low numbers of satellite cells that proliferate longer contributing to poor myofiber regeneration and higher deposit of collagen [13]. Therefore, it could be inferred that suture techniques which predisposed to prolonged inflammation in limb muscle would intensify scar formation in orofacial muscles.

## 2. Methods

### 2.1. Animal Model

**Figure 1:**
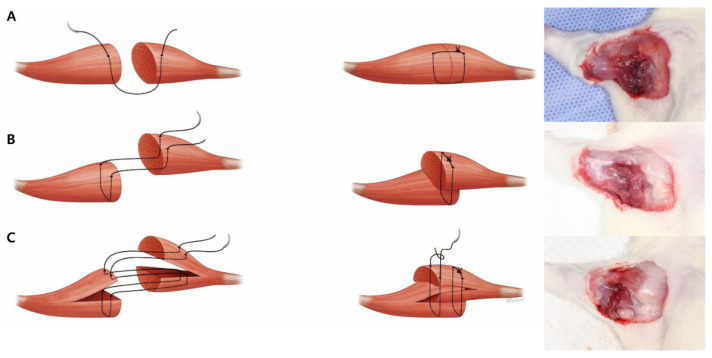
Muscle suture techniques. (**A**) Simple interrupted sutures. (**B**) Overlapping sutures. (**C**) Splitting-interdigitating sutures.

A total of thirty adult male Sprague–Dawley (SD) rats (Orient Bio, Inc., Seongnam, Korea) weighing 230 to 260 g were used in this study. All experimental procedures conducted in the study were approved by The University Animal Care Committee for Animal Research of Keimyung University. The animals were anesthetized via intraperitoneal injection of a mixture of zolazepam/tiletamine (30 mg/kg, Zoletil^®^; Virbac, Carros, France) and xylazine (10 mg/kg, Rompun^®^; Bayer, Leverkusen, Germany). A small linear incision was made in the right axillary skin. The triceps muscle was exposed via blunt dissection and cut horizontally along the midline using a no. 15 blade. Then, the muscle was repaired using Vicryl 4-0 (Ethicon, Inc, Raritan, NJ, USA) with three different suture techniques: simple interrupted sutures (simple group, *n* = 10), overlapping sutures (overlapping group, *n* = 10), and splitting-interdigitating sutures (splitting group, *n* = 10; Figure 1).

### 2.2. Grip Strength Test

**Figure 2:**
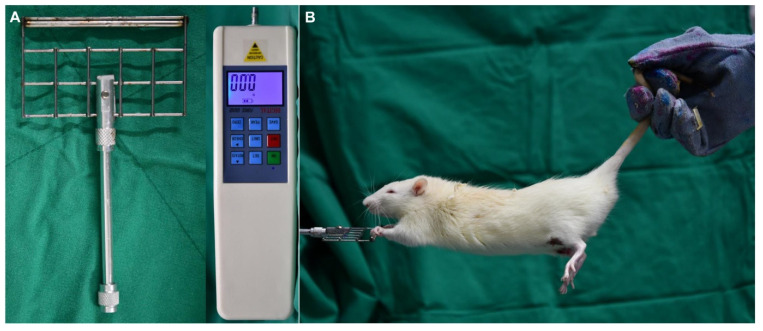
(**A**) Digital force transducer. (**B**) The grip strength test was repeated twice, and the average of the measured values was calculated.

Forelimb grip strength was measured bilaterally using the grasping test as described by Bertelli and Mira [14]. The animal was held by its tail, allowed to grip a rigid bar, and then slowly pulled upward until it released its grip. A customized aluminum grip bar was mounted to a force transducer connected to a digital display and recording unit (digital force gauge HF-3000^®^; Shenzhen Aermanda Technology, Guangdong, China). The test was repeated twice, and the average grip force per weight (N/100 g) was used for the statistical analysis. The person carrying out the testing was blinded to the measurements (Figure 2). The 5 SD rats per group that were not sacrificed for biopsy at week 1 were subjected to the grip strength, which was performed before surgery to obtain baseline measurements and at postoperative weeks 1, 2, 4, and 8.

### 2.3. Spatial Gait Symmetry Test

**Figure 3:**
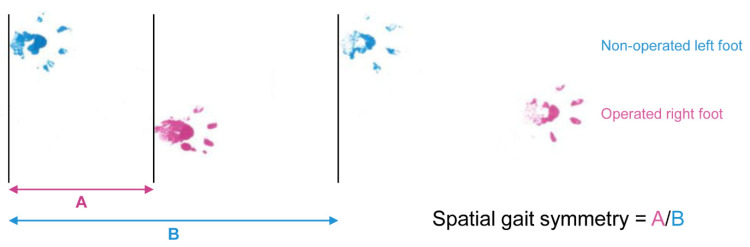
Spatial gait symmetry test.

A gait test was performed to analyze range of motion by comparing the strides of the forepaw on the operated side with those of the forepaw on the nonoperated side. First, the right forepaw was painted with a thin layer of water-soluble pink dye, and the left forepaw was painted blue. Each rat was placed at the open end of a passageway that was 60 cm in length and covered with a sheet of clear plastic and allowed to proceed to the opposite end in the absence of any external stimuli. As the rat voluntarily proceeded through the passageway, its steps were recorded on an underlying sheet of paper. Spatial symmetry was calculated as the ratio of the step length (distance from the left forepaw to the right forepaw) to the stride length (distance from the left forepaw to next left forepaw) (Figure 3). A spatial symmetry of approximately 0.5, which suggested that the right foot strikes (operated side) were spatially centered between two left foot strikes (nonoperated side), indicated a spatially symmetrical gait. A spatially nonsymmetrical gait indicates that the rat may have problems stretching the right forelimb or shifting its weight from the right forelimb to the left forelimb. The 5 SD rats per group that were not sacrificed for biopsy at week 1 were subjected to the spatial gait symmetry test at postoperative weeks 1, 2, 4, and 8.

### 2.4. Histological Analysis

After 5 SD rats per group were sacrificed in a carbon oxide chamber at postoperative weeks 1 and 8, the triceps muscles were isolated for histological and immunohistochemical examination.

The specimens were fixed in formalin, embedded in paraffin, and sectioned into 5-μm-thick slices. Standard hematoxylin and eosin (H&E) staining was performed to evaluate the inflammatory cell response and general morphological features. The tissue samples were observed using a light microscope. All slides were reviewed by pathologists blinded to the study conditions.

### 2.5. Western Blot Analysis

Rabbit polyclonal antibodies against IL-1 (Santa Cruz Biotechnology, Dallas, TX, USA) and TNF-α (Abcam, Cambridge, UK) were used as primary antibodies; the expression of glyceraldehyde-3-phosphate dehydrogenase (GAPDH), a housekeeping gene, was also measured with a specific antibody. Immunoreactive bands were detected by image scanning using a Fusion FX system (Vilber, Collégien, France).

### 2.6. Immunohistochemistry

Formalin-fixed, paraffin-embedded, 5-μm-thick tissue sections were subjected to automated immunohistochemistry with a Ventana BenchMark XT immunostainer (Ventana Medical Systems, Oro Valley, AZ, USA). The sections were incubated with a mouse anti-TNF-α antibody (Abcam, Cambridge, UK) diluted 1:10,000 and a mouse anti-IL-1β antibody (Santa Cruz Biotechnology, Dallas, TX, USA) diluted 1:5000 for 32 min and then visualized with an Optiview DAB IHC Detection Kit (Ventana Medical Systems, Oro Valley, AZ, USA) following amplification of the signal. The specimens were counterstained with a Ventana kit (hematoxylin for 4 min and Bluing Reagent for 4 min).

To detect regenerating muscle fibers, fluorescence immunohistochemistry was performed with anti-laminin (Novus Biologicals, CO, USA) and anti-MyoD antibodies (Santa Cruz Biotechnology, Dallas, TX, USA). Each specimen was also stained with DAPI to visualize the nuclei of mitotic cells. Immunohistochemistry was carried out using typical methods.

### 2.7. Statistical Analysis

Spatial gait symmetry and grip strength were statistically analyzed using two-way ANOVA with GraphPad Prism 8^TM^ (GraphPad Software, Inc., San Diego, CA, USA). Differences with a value of *p* < 0.05 were considered statistically significant.

## 3. Results

### 3.1. The Simple Group and Overlapping Group Exhibit Higher Muscle Power Than, but a Similar Range of Motion as the Splitting Group

**Figure 4:**
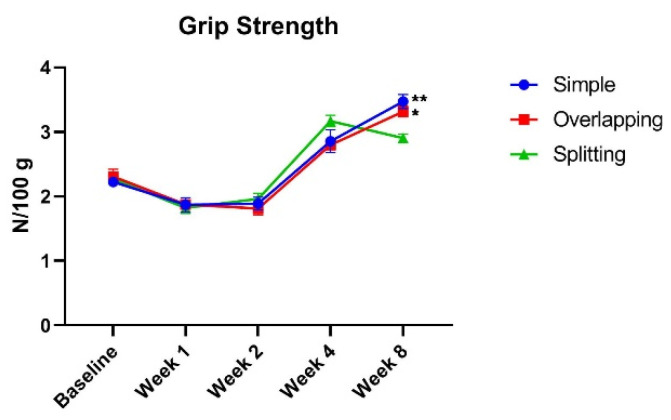
Grip strength test. Although all groups demonstrated the same grip strength at baseline, the simple interrupted suture and overlapping suture groups demonstrated significantly higher muscle power than the splitting suture group at week 8 (simple vs. splitting, ** *p* = 0.0092; overlapping vs. splitting, * *p* = 0.0113).

A reduction in grip force (N/100 g) was observed by postoperative week 2 regardless of suture technique. Given that grip force per weight gradually increases as rats grow, the rats seemed to achieve a normal level of grip strength at week 4. However, there was no significant difference in grip strength between the three groups until week 4. At week 8, the grip force per weight of the simple group (median, 3.49; IQR, 3.28 to 3.66) and overlapping group (median, 3.3; IQR, 3.17 to 3.47) was significantly higher than that of the splitting group (median, 2.91; IQR, 2.76 to 3.05), as determined by two-way ANOVA (Figure 4). This finding suggests that simple and overlapping sutures induce better physiological recovery than splitting sutures.

**Figure 5:**
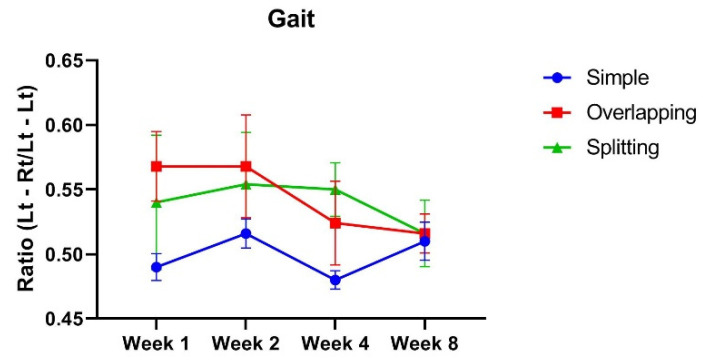
Gait test. The overlapping and splitting suture groups demonstrated asymmetrical gait intervals between the left and right sides through week 4. However, there was no significant difference in gait intervals between any of the groups, and the difference in gait was similar between all groups at week 8.

The spatial gait symmetry test was used to evaluate the geometrical pattern of foot strikes. Whereas the gait of the simple group was almost symmetric, with a median value of 0.5 (IQR, 0.47 to 0.51), the gaits of the overlapping and splitting groups were spatially asymmetric, with values of 0.55 (IQR, 0.53 to 0.62) and 0.51 (IQR, 0.45 to 0.63), respectively. Over time, the gait symmetry values of all groups became closer to 0.5 as muscle healing progressed, with the simple group exhibiting a value of 0.52 (IQR, 0.48 to 0.54), the overlapping group showing a value of 0.52 (IQR, 0.51) and the splitting group exhibiting a value of 0.51 (IQR, 0.47 to 0.57) at week 8 (Figure 5). However, there was no statistical significance between any of the groups during the experimental period. This finding indicates that the suture technique did not influence the extent of muscle contraction.

### 3.2. Simple Suturing Leads to Earlier Remission of Inflammation Than Overlapping or Splitting Suturing

**Figure 6:**
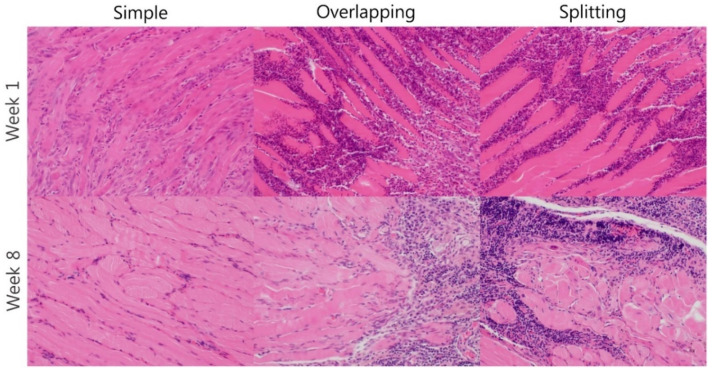
H&E staining. The overlapping and splitting groups exhibited prolongation of infiltration of inflammatory cells into interstitial tissue until week 8. In contrast, inflammatory infiltration was resolved in the simple interrupted suture group at week 8.

H&E staining revealed significant infiltration of inflammatory cells into the area between muscle fibers in all groups at week 1. At week 8, the overlapping and splitting groups still demonstrated marked infiltration of inflammatory cells into the interstitial tissue between muscle fibers. However, significant remission of inflammation was observed in the simple group compared with the overlapping and splitting groups (Figure 6).

**Figure 7:**
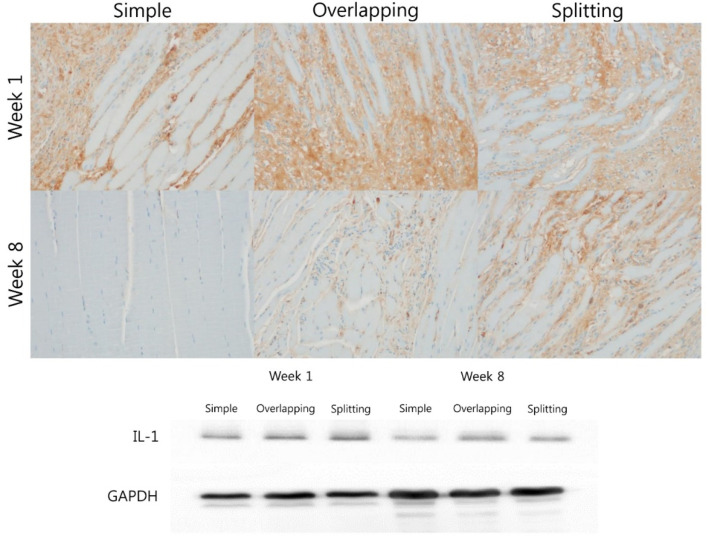
Immunohistochemical and Western blot analysis of IL-1 expression. At week 1, all groups showed strong expression of IL-1 by immunohistochemistry and Western blotting. The simple interrupted suture group exhibited weaker expression of IL-1 than the overlapping and splitting suture groups by immunohistochemistry and Western blotting.

IL-1β and TNF-α are cytokines that are important mediators of the acute inflammatory response. At week 1, IL-1 expression between muscle fibers was high in all groups. IL-1 expression was almost eliminated in the simple and overlapping groups at week 8, but the splitting group exhibited stronger expression of IL-1 than the overlapping and simple groups. Western blot analysis of IL-1 revealed similar temporal changes. Although IL-1 expression was similarly high in all groups at week 1, the simple group demonstrated lower expression of IL-1 than the overlapping and splitting groups at week 8 (Figure 7).

**Figure 8:**
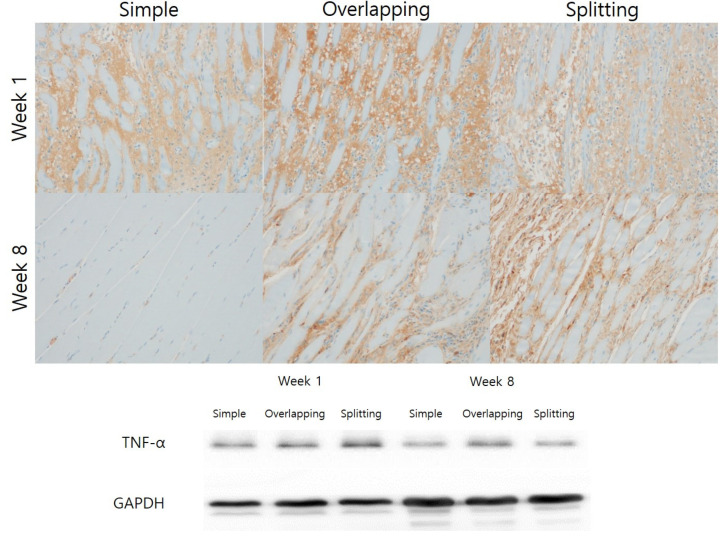
Immunohistochemical and Western blot analysis of TNF-α expression. At week 1, all groups showed strong expression of TNF-α in interstitial tissue by immunohistochemistry. The splitting suture group exhibited higher TNF-α levels than the overlapping and simple interrupted suture groups by Western blotting. The simple interrupted suture group exhibited weaker expression of TNF-α than the other groups by immunohistochemistry and Western blotting at week 8.

Immunohistochemistry and Western blotting showed that TNF-α expression was higher in the overlapping and splitting suture groups than in the simple group at week 1. At week 8, immunohistochemistry and Western blotting revealed that TNF-α expression was abolished in the simple group. Immunohistochemistry and Western blotting revealed that TNF-α expression in the simple group was significantly lower at week 8 than at week 1, while the overlapping and splitting groups showed higher TNF-α expression at week 8 (Figure 8).

### 3.3. Simple and Overlapping Suturing Induced Organized Remodeling of Healing Muscle at Week 8

**Figure 9:**
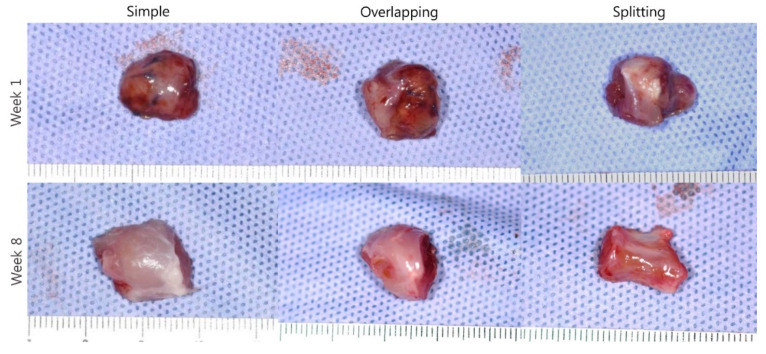
Gross examination.

Overlapping and splitting suturing demonstrated good preservation of the muscle shape initially after suturing and a thicker muscle belly compared with simple suturing on gross examination at week 1. However, splitting suturing demonstrated an atrophied muscle belly at week 8 in contrast to the muscle belly that was similar in thickness in simple and overlapping suturing (Figure 9).

**Figure 10:**
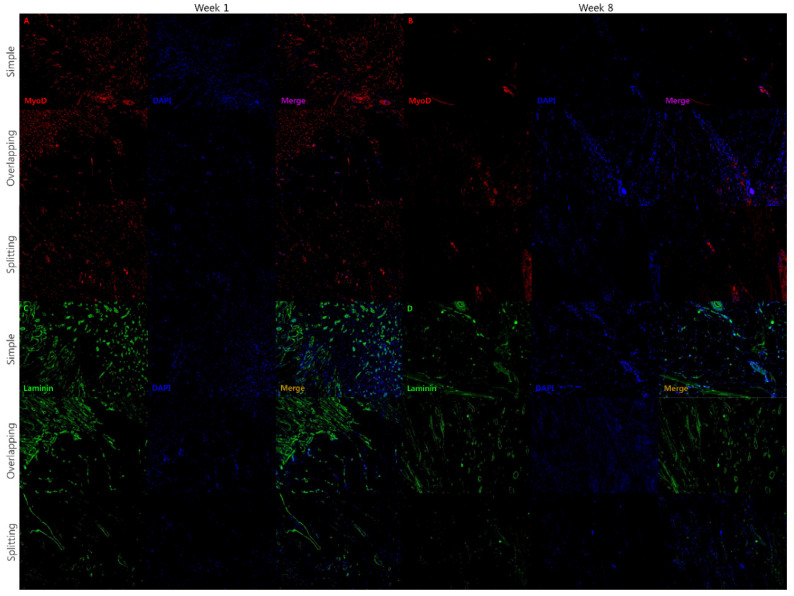
Immunofluorescence for MyoD and laminin. (**A**) Muscle regeneration was strongly activated, as evidenced by strong expression of MyoD, in all groups at week 1. (**B**) The simple interrupted suture group exhibited lower muscle regeneration activity and MyoD expression than the overlapping and splitting suture groups, in which the expression of MyoD was maintained at week 8. (**C**) The simple interrupted suture and overlapping suture groups exhibited organized muscle remodeling with strong expression of laminin at weeks 1 and 8. (**D**) In contrast, the splitting suture group showed weak expression of laminin, indicating unorganized and scarce muscle remodeling, until week 8.

MyoD is expressed in the regenerative phase of muscle healing, whereas laminin is a marker of mature skeletal muscle. At week 1, the expression of MyoD at the muscle suture site was similarly high in all groups. MyoD expression was significantly lower in the simple group at week 8 than at week 1, whereas MyoD expression persisted in the overlapping and splitting groups at week 8 (Figure 10). The regenerative phase of muscle wound healing ceased in the simple group by week 8, while the overlapping and splitting groups demonstrated prolonged muscle healing at week 8. At week 1, the simple group demonstrated significantly higher laminin expression at the muscle suture site than the overlapping and splitting groups. At week 8, The simple group and overlapping groups demonstrated equivalent expression of laminin at the muscle suture site. However, the splitting group demonstrated low expression of laminin at the muscle suture site during the experimental period (Figure 10). This finding indicates that the splitting group exhibited prolongation of inflammation and the regenerative phase followed by poor muscle fiber remodeling.

## 4. Discussion

Our results show that simple sutures demonstrated less inflammation and more favorable histological healing than splitting-interdigitating sutures. Simple and overlapping sutures demonstrated similar functional healing, although greater inflammation and failure to maintain a thicker muscle belly were observed in the overlapping suture group compared with the simple suture group.

Muscle healing occurs in five time-dependent phases: degeneration, inflammation, regeneration, remodeling, and functional recovery [15]. Injured muscle fibers release intracellular components that trigger inflammatory reactions [16]. Neutrophils and mast cells initially recruit inflammatory cells to injured muscle. Degranulation of resident mast cells causes the release of proinflammatory cytokines, such as TNF-α and IL-1 [17]. These cytokines recruit neutrophils to the lesion, which induces phagocytosis of necrotic debris. Then, quiescent satellite cells of the basal lamina are activated and differentiated into myoblasts, which fuse with existing myofibers and repair damaged muscle fibers [15,18]. Activated satellite cells express myogenic biomarkers, such as Pax7, Mcad, VCAM1 and MyoD, during the regenerative phase [19,20]. The major structural protein of the basal lamina, laminin, is found in mature skeletal muscle and ECM networks [21]. Based on the sequence of muscle wound healing, we analyzed TNF-α and IL-1 expression to evaluate the extent of inflammation, MyoD expression to determine the degree of muscle healing, and laminin expression to assess muscle remodeling. Furthermore, we analyzed functional recovery based on muscle strength and range of motion.

**Figure 11:**
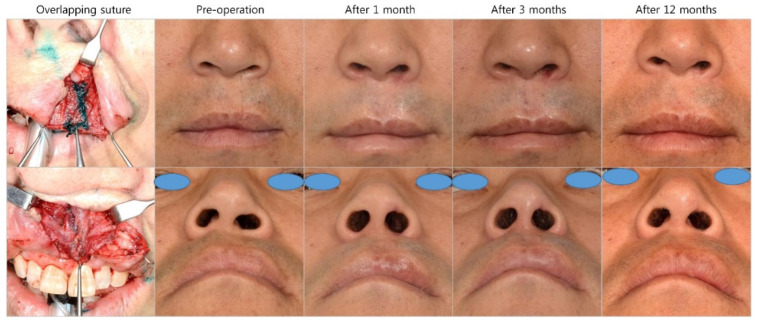
Case of overlapping suture technique. A 44-year-old man underwent secondary correction of cleft lip nasal deformity using overlapping suturing of the OOM. A 5-mm overlap of the OOM from the lateral lip to the medial lip was performed. Augmentation of the philtral column and nasal base was maintained until 3 months after the operation, However, relapse resulting in a vague philtral column and depression of the nasal base was observed 12 months after the surgery.

Overlapping suturing of the OOM has been recognized as an effective method for mimicking philtral columns in cleft lip repair [6]. Chang et al. [6] reported that overlapping mattress sutures form better philtral columns than asymmetrical edge-to-edge sutures based on three-dimensional photographic anthropometry and ultrasonographic measurements. Indeed, the operators anticipated that overriding the OOM would maintain the stepped suturing shape to mimic the philtral column as a “flaked ridge”. Although they reported that overlapping sutures demonstrated a favorable philtral column shape compared with the asymmetrical edge-to-edge suture technique, we wondered whether the sutured muscle would maintain its shape on gross and histological analysis because muscle is continuously acting tissue. In fact, interrupted sutures result in the healing of thicker muscles and stronger resistance to tension than overlapping sutures [11]. In concordance with a previous study, our results also demonstrated that simple interrupted sutures demonstrated higher muscle strength, a weaker inflammatory reaction and better muscle healing upon histological analysis than the other suture techniques. Overlapping sutures led to a similar muscle thickness, strength, and range of motion as the simple interrupted, but with a stronger inflammatory reaction. Indeed, the purpose of overlapping sutures is the construction of thicker muscle to mimic the ridge of philtral column in cleft lip surgery and more powerful closure of the LVP muscle in palatoplasty. However, we did not observe that overlapping sutures had additional effects on muscle thickness or muscle healing. Furthermore, the splitting-interdigitating suture technique led to unfavorable outcomes related to muscle strength, the inflammatory response, and muscle healing. The majority of modifications to the OOM suture technique in cleft lip surgery involving the splitting of both sides or one side of the OOM following by interdigitating, folding or vertical mattress suturing [7,8,9,22]. The splitting procedure may induce additional muscle injury and inflammation that hinder muscle healing, as observed by the significantly lower muscle strength in the splitting-interdigitating group than the simple interrupted suture group in our study. We speculate that additional manipulation of muscle tissue could induce muscle injury, ischemia, and poor healing. We also frequently used the overlapping suture technique to reconstruct the philtral column and augment the nasal base. In our experience, overlapping sutures demonstrated a distinct philtral column shape and augmented the nasal base immediately after the operation. However, we experienced some relapse of collapse of the philtral column and nasal base despite the use of overlapping sutures (Figure 11). Therefore, it is necessary to apply additional procedures with overlapping sutures to reconstruct the philtral column. In fact, adjunctive procedures have been reported in reconstruction of the philtral column using several grafts, such as concha cartilage, dermal, and tendon grafts [23,24,25].

One limitation of our study is that we used skeletal muscle rather than facial mimetic muscle. Fundamentally, the origin of satellite cells, which are key cells for muscle regeneration, is different between orofacial muscle and skeletal muscle. Satellite cells of orofacial muscle originate from the mesoderm, while satellite cells of skeletal muscle originate from the mesoderm or ectoderm [26]. Interestingly, soft palate muscle regeneration involves higher collagen deposition and less myofiber regeneration than skeletal muscle regeneration [27]. Furthermore, satellite cells from orofacial muscle differentiate later than those from skeletal muscle [13]. Orofacial muscle regeneration involves more scar-forming healing than skeletal muscle regeneration. Based on these known differences, wound healing process in orofacial muscle is predictable. Thus, a suture technique that results in the formation of more scar tissue in skeletal muscle could intensify scar formation in orofacial muscle. Therefore, muscle regeneration following overlapping and splitting-interdigitating suturing could be impaired in an orofacial muscle model. Although models of orofacial muscle injury are available, the majority of these models are muscle defect models that cannot be used to evaluate suture techniques or for functional analysis [27,28,29]. Therefore, we assessed skeletal muscle in this study. In the future, we will strengthen our results by performing a human study involving imaging or a larger animal study using orofacial muscle.

There are various techniques used to suture the OOM in cleft lip surgery. Overlapping sutures and modified suture techniques, including splitting sutures, are popular suture methods used by cleft surgeons to mimic the philtral column or strengthen the muscle. Nevertheless, simple interrupted sutures and overlapping sutures showed similar muscle healing in this study. Therefore, there is some limitation in reconstructing the philtral column using overlapping sutures alone, and additional procedures could be needed.

## Data Availability

The data presented in this study are available on request from the corresponding author.

## Abbreviations

H&E, hematoxylin and eosin; OOM, orbicularis oris muscle; LVP, levator veli palatine; GAPDH, glyceraldehyde-3-phosphate dehydrogenase.
